# Metasurfaces Assisted Twisted α-MoO_3_ for Spinning Thermal Radiation

**DOI:** 10.3390/mi13101757

**Published:** 2022-10-17

**Authors:** Yasong Sun, Derui Zhang, Biyuan Wu, Haotuo Liu, Bing Yang, Xiaohu Wu

**Affiliations:** 1 Basic Research Center, School of Power and Energy, Northwestern Polytechnical University, Xi’an 710072, China; 2 Center of Computational Physics and Energy Science, Yangtze River Delta Research Institute of NPU, Northwestern Polytechnical University, Taicang 215400, China; 3 Shandong Institute of Advanced Technology, Jinan 250100, China; 4 School of Energy Science and Engineering, Harbin Institute of Technology, Harbin 150001, China; 5 Centre for Advanced Laser Manufacturing (CALM), School of Mechanical Engineering, Shandong University of Technology, Zibo 255000, China

**Keywords:** spin thermal radiation, metasurface, twisted α-MoO_3_

## Abstract

Spinning thermal radiation has demonstrated applications in engineering, such as radiation detection and biosensing. In this paper, we propose a new spin thermal radiation emitter composed of the twisted bilayer α-MoO_3_ metasurface; in our study, it provided more degrees of freedom to control circular dichroism by artificially modifying the filling factor of the metasurface. In addition, circular dichroism was significantly enhanced by introducing a new degree of freedom (filling factor), with a value that could reach 0.9. Strong-spin thermal radiation resulted from the polarization conversion of circularly polarized waves using the α-MoO_3_ metasurface and selective transmission of linearly polarized waves by the substrate. This allowed for extra flexible control of spinning thermal radiation and significantly enhanced circular dichroism, which promises applications in biosensing and radiation detection. As a result of their unique properties, hyperbolic materials have applications not only in spin thermal radiation, but also in areas such as near-field thermal radiation. In this study, hyperbolic materials were combined with metasurfaces to offer a new idea regarding modulating near-field radiative heat transfer.

## 1. Introduction

In recent years, thermal radiation has attracted considerable attention from researchers due to its high potential for applications in areas such as energy harvesting [1,2,3,4] and coherent heat sources [5,6,7]. According to wave-particle duality, the nature of thermal radiation is electromagnetic waves. Therefore, thermal radiation possesses various properties of electromagnetic waves, such as superposition and coherence properties, spectral properties and polarization properties [8,9,10]. Greffet et al. demonstrated that periodic microstructures could emit a coherent and linearly polarized wave [5], which offers significant promise for controlling the spectral, coherent and polarization properties of thermal radiation [11,12,13]. Spin polarized (circularly polarized) wave has gained extensive attention in chiral optics [14,15,16] and spin-controlled nanophotonics [17,18,19]; spin angular momentum is used to engineer spin-dependent nanoscale light-matter interactions. Recently, studies regarding chiral microstructures have demonstrated the feasibility of spin thermal radiation for engineering, including thermal detection [20,21,22].

In general, spin thermal radiation can be generated by breaking rotational symmetry and mirror symmetry simultaneously. Circular dichroism (CD) is defined as the difference in the absorption between left-hand circular polarization (LCP) and right-hand circular polarization (RCP); CD is an important parameter when measuring spin thermal radiation [21,22]. At present, many approaches have been proposed to improve CD [23,24]. It is possible to break mirror symmetry using an applied magnetic field (due to the spin-orbit interaction of electrons) resulting in spin thermal radiation [25]. Nevertheless, this approach requires additional incentives and is not conducive to practical application.

Hyperbolic materials (HMs) have attracted much attention due to their unique properties [26,27]. HMs have a wide range of promising applications in broadband enhanced local density of states (LDOS) [28], spontaneous emission [29,30,31], hyperbolic lensing [32,33,34], negative refraction [35,36], super absorption [37] and Förster energy transfer [38,39,40]. As a natural biaxial hyperbolic crystal with in-plane anisotropy, α-MoO_3_ has a unique advantage in exciting spin thermal radiation. Hexagonal boron nitride (hBN) is another hyperbolic material with out-of-plane anisotropy, which is also capable of exciting spin thermal radiation. Generally, spin thermal radiation requires more anisotropy. Compared to the uniaxial hyperbolic material hBN, α-MoO_3_ is a natural biaxially hyperbolic material with both in-plane and out-of-plane anisotropy, enabling it to facilitate spin thermal radiation. In addition, α-MoO_3_ has a wider hyperbolic band, carrying larger electromagnetic wave energy, which offers the possibility of enhancing circular dichroism. [41]. Wu et al. studied the spin thermal radiation properties of single-layer α-MoO_3_ [42] and double-layer twisted α-MoO_3_ structures [43]. Although the structures mentioned above can excite spin thermal radiation properties, the CD obtained by optimizing the rotation angle and thickness parameters was always very limited.

Another way to achieve spin thermal radiation is to create a structure with chiral surface morphology or with the help of chiral metamaterials. Dyakov et al. proposed a photonic crystal slab waveguide with chiral morphology that can excite spin thermal radiation without an external magnetic field [44]. Kong et al. proposed a novel chiral metamaterial structure with Γ-shaped aligned nanocrystals to achieve significant CD [24]. To date, many two-dimensional (2D) or three-dimensional (3D) chiral microstructures have been designed that enhance spin thermal radiation significantly [45,46]. Although chiral metamaterials can effectively improve CD, subwavelength nanostructures tend to increase the complexity of structural fabrication. Metasurfaces, as two-dimensional derivatives of metamaterials composed of a single or a few patterned layer planar structures, reduce the fabrication requirement. In recent years, metasurfaces have attracted much attention from researchers and have a high potential for important applications [47,48]. More importantly, thermal radiation devices based on metasurfaces possess more freedom of regulation. Recently, metasurfaces based on α-MoO_3_ rectangular strips, which only need to be etched on a single layer of slab, have attracted interest. Huang et al. [49] studied hyperbolic phonon polarization excitons (HPhPs) of van der Waals semiconductors coupled to terahertz and LWIR radiation based on gratings etched directly on α-MoO_3_ semiconductor flat plates, ultimately obtaining quality factors as high as 300. However, the spin thermal radiation of α-MoO_3_ microstructures is still seldom studied.

This paper describes our study of the spin thermal radiation properties of the metasurface-assisted twisted bilayer α-MoO_3_. First, the effects of the thicknesses of the two layers and the rotation angle on the CD value were investigated. In addition, a new degree of freedom (filling factor) was introduced. It was found that the structure can greatly enhance spin thermal radiation, and also provide more degrees of freedom to control the spin thermal radiation instead of limiting it to a specific angle. Furthermore, this paper explains the physical mechanism of CD dependence on the filling factor from the perspective of polarization conversion. This study achieved strong spin thermal radiation, which allows greater freedom in tuning the spin thermal radiation.

## 2. Theory and Method

**Figure 1:**
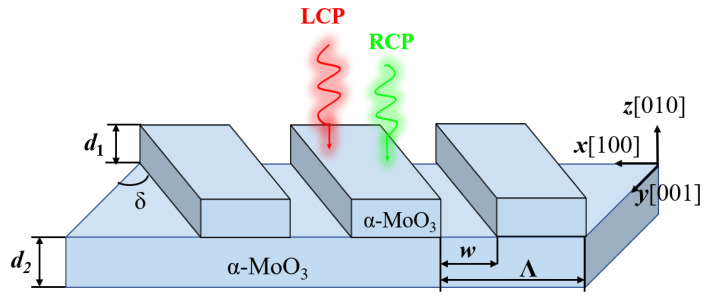
The metasurface structure with spin thermal radiation; both substrate and rectangular strips are α-MoO_3_.

Figure 1 shows the proposed metasurface structure, which consists of a periodic α-MoO_3_ rectangular strip and an α-MoO_3_ substrate. As shown in Figure 1, *d*_1_ and *d*_2_ represent the thicknesses of rectangular strips and substrate, respectively. δ represents the relative rotation angle between the rectangular strips and the substrate. When the rectangular strips had a rotation angle with respect to the substrate, the overall symmetry of the structure broke. *w* represents the spacing of the rectangular strips, Λ is the period, and the incident light was directed along the *z*-axis. For the α-MoO_3_ substrate, the crystal axes [100], [001] and [010] were along the *x*, *y* and *z* directions, respectively. Thus, the permittivity tensor of the α-MoO_3_ substrate can be denoted by $\varepsilon =diag(\varepsilon_{x},\varepsilon_{y},\varepsilon_{z})$, where $\varepsilon_{x}$, $\varepsilon_{y}$ and $\varepsilon_{z}$ can be represented by the Lorentz model as [50]:

(1) $$\varepsilon_{m}=\varepsilon_{\infty ,m}\left(1+\frac{\omega_{LO,m}^{2}-\omega_{TO,m}^{2}}{\omega_{TO,m}^{2}-\omega^{2}-j\omega \Gamma_{m}}\right)$$

where *w* is the angular frequency. The values of the other parameters are shown in Table 1 [51].

We first analyzed the top α-MoO_3_ rectangular strips using the effective medium theory [52]. The effective permittivity can be expressed as:

(2) $$\begin{array}{l} \varepsilon_{eff,xx}=({\frac{f}{\varepsilon_{\alpha -MoO_{3},x}}+1-f)}^{-1}\\ \varepsilon_{eff,yy}=\varepsilon_{\alpha -MoO_{3},y}f+1-f\\ \varepsilon_{eff,zz}=\varepsilon_{\alpha -MoO_{3},z}f+1-f \end{array}$$

where *f* is the filling factor and its value is *f* = *w*/Λ.

When the top rectangular strips had a rotation angle δ with respect to the substrate, rotation broke the diagonal tensor form of the original dielectric function; the permittivity tensor of α-MoO_3_ follows the following transformation form [53]:

(3) $$\varepsilon =\left(\begin{array}{ccc} \cos\delta & -\sin\delta & 0\\ \sin\delta & \cos\delta & 0\\ 0 & 0 & 1 \end{array}\right)\left(\begin{array}{ccc} \varepsilon_{\mathrm{eff},xx} & 0 & 0\\ 0 & \varepsilon_{\mathrm{eff},y} & 0\\ 0 & 0 & \varepsilon_{\mathrm{eff},z} \end{array}\right)\left(\begin{array}{ccc} \cos\delta & \sin\delta & 0\\ -\sin\delta & \cos\delta & 0\\ 0 & 0 & 1 \end{array}\right)$$

The new permittivity tensor was obtained after the calculation as follows:

(4) $$\varepsilon =\left[\begin{array}{ccc} \varepsilon_{eff,xx}cos^{2}\delta +\varepsilon_{eff,yy}\sin^{2}\delta & \left(\varepsilon_{eff,xx}-\varepsilon_{eff,yy}\right)\sin\delta \cos\delta & 0\\ \left(\varepsilon_{eff,xx}-\varepsilon_{eff,yy}\right)\sin\delta \cos\delta & \varepsilon_{eff,xx}\sin^{2}\delta +\varepsilon_{eff,yy}\cos^{2}\delta & 0\\ 0 & 0 & \varepsilon_{eff,zz} \end{array}\right]$$

In this study, the transfer matrix method (TMM) was used to calculate the transmission of the above structures [43].

A large area of α-MoO_3_ flakes was first grown using the physical vapor deposition method. This was then transferred to a silicon substrate and a combination of electron beam lithography and reactive ion etching was used to etch one-dimensional nanoribbons with different periods and angles on the flakes. Electron beam lithography was performed using a Poly (methyl methacrylate) (PMMA) photoresist and ion etching was performed using a mixture of oxygen, argon and CHF_3_ at 50 W for 10 min, after which we obtained the α-MoO_3_ 1D grating structure [49].

## 3. Results and Discussion

CD is a key parameter for measuring spin thermal radiation’s radiative properties. In this study, we primarily considered the transmission of the structure. Therefore, CD could be calculated using:

(5) $$CD=\left|T_{LCP}-T_{RCP}\right|,$$

where $T_{LCP}$ and $T_{RCP}$ are the transmission of the LCP and RCP waves, respectively.

**Figure 2:**
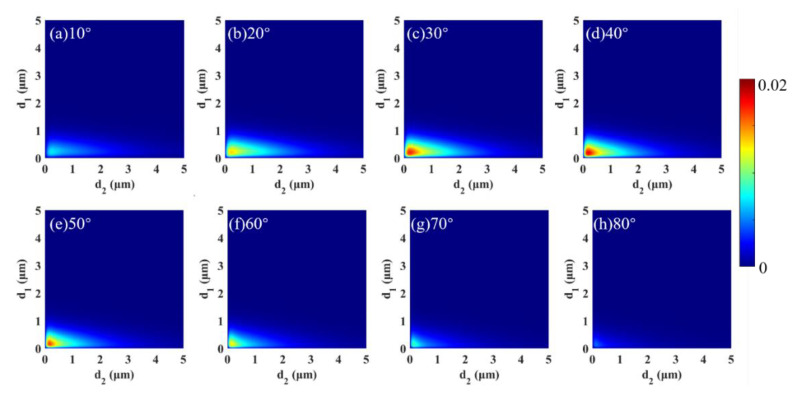
When the wavelength was fixed at 12 μm, and *f* = 0, CD varied with *d*_1_ and *d*_2_ for different rotation angles: (**a**) 10°, (**b**) 20°, (**c**) 30°, (**d**) 40°, (**e**) 50°, (**f**) 60°, (**g**) 70° and (**h**) 80°.

Based on [44], it is known that the thickness and the relative rotation angle significantly influence the spin radiation properties of the structure. The variation in CD with thickness and the relative rotation angle was first calculated for any wavelength (here, the wavelength was fixed at 12 μm) and *f* = 0 (bilayer slabs), as shown in Figure 2. The CD value tended to increase and then decrease as the angle of rotation increased. CD reached a maximum value of 0.0178 at *d*_1_ = *d*_2_ = 0.175 μm. Although CD can be controlled by changing the rotation angle, the CD was still very weak. Results indicate that there was almost no excitation of spin thermal radiation at *f* = 0; therefore, the bilayer slabs had some limitations regarding exciting spin thermal radiation.

**Figure 3:**
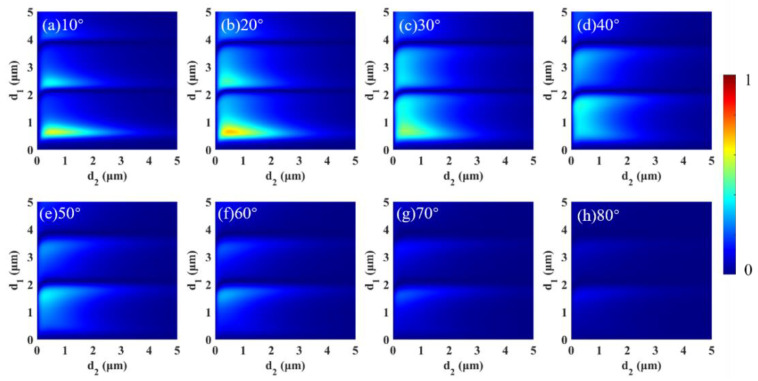
When the wavelength was fixed at 12 μm and *f* = 0.1, CD varied with *d*_1_ and *d*_2_ for different rotation angles: (**a**) 10°, (**b**) 20°, (**c**) 30°, (**d**) 40°, (**e**) 50°, (**f**) 60°, (**g**) 70° and (**h**) 80°.

Based on the above study, we introduced the filling factor *f*. Next, the effect of *f* on CD is discussed in detail. Here, the wavelength was the same as that in Figure 2. Variation in CD with *d*_1_ and *d*_2_ as well as the rotation angle are provided in Figure 3. Here, the grating period of the grating was 3 μm. Notably, the maximum value of the color bar is 1, whereas that of Figure 2 is 0.02. Compared to when *f* = 0, CD has been significantly enhanced. CD could reach 0.6848 at *d*_1_ = 0.65 μm, *d*_2_ = 0.525 μm and a 20° rotation angle, which is tens of times higher than that at *f* = 0. The results illustrate that the metasurface structure greatly enhanced spin thermal radiation. In addition, we used the same method to optimize the structure; it was found that CD could reach 0.9 when *f* = 0.7, *d*_1_ = 6.25 μm, *d*_2_ = 0.5 μm and δ = 40°, which exceeded the results in previous studies [45,46].

**Figure 4:**
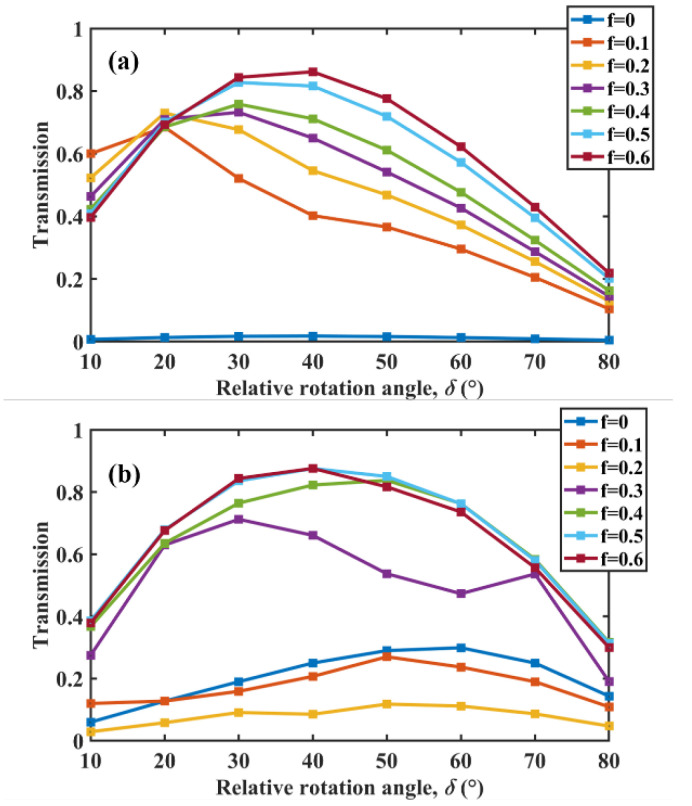
Maximum value of CD as a function of the rotation angle under different *f* when the wavelength was (**a**) 12 μm and (**b**) 11 μm, respectively.

Next, to further illustrate the effect of *f* on CD, we calculated the variation in the maximum value of CD with the rotation angle when *f* increased from 0 to 0.6 at every 0.1 interval. Figure 4a,b show results for wavelengths of 12 μm and 11 μm, respectively. In Figure 4a, it can be seen that the overall trend of CD increased with an increase in *f*, implying that the value of *f* can enhance the spin thermal radiation in a wide range, which is more beneficial to practical applications. When the wavelength was 11 μm, it can be seen in Figure 4b that, although the CD decreased somewhat at *f* = 0.1 and *f* = 0.2, it still showed an overall increasing trend at larger *f*. We conducted similar studies at other wavelengths, with results similar to those of 12 μm and 11 μm, namely that CD was enhanced as *f* increased. This suggests that the metasurface structure not only enhances spin thermal radiation, but also has a greater degree of freedom in the excitation of thermal radiation.

**Figure 5:**
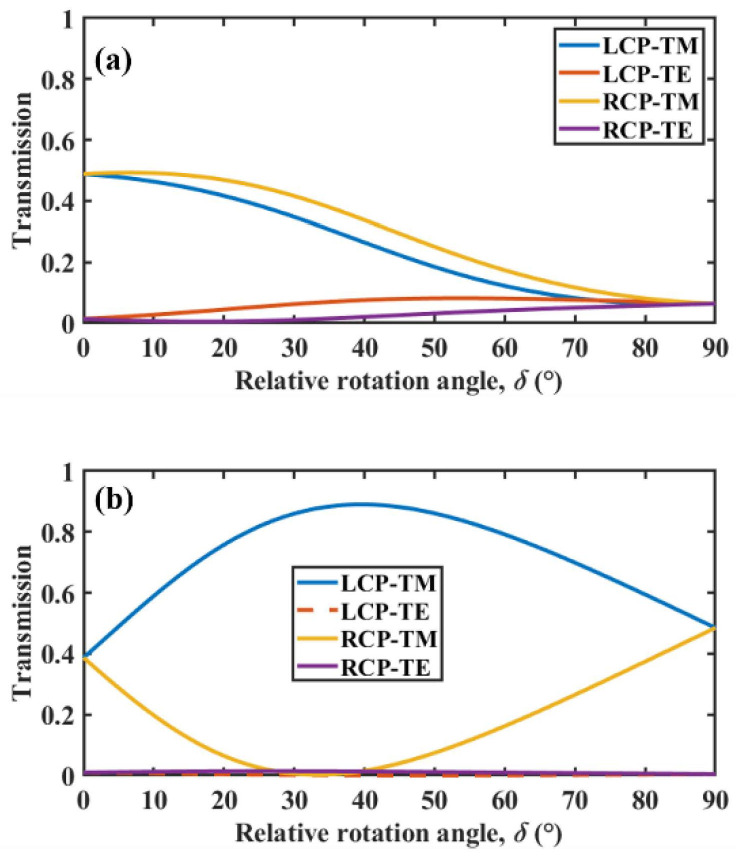
TE wave and TM wave components in the transmitted wave as a function of the rotation angle for LCP and RCP waves: (**a**) *f* = 0 and (**b**) *f* = 0.6.

To better understand the physical mechanism, we discuss the polarization conversion of circularly polarized waves at a fixed wavelength of 12 µm. Figure 5a shows TE (transverse electric wave) and TM (transverse magnetic wave) components in the transmitted wave varying with the rotation angle for different spin direction circularly polarized waves incidence when *f* = 0 and *d*_1_ = *d*_2_ = 0.175 μm. LCP-TM represents the TM wave component in the transmitted wave for LCP wave incidence; RCP-TM, RCP-TE and LCP-TE have similar definitions. When *f* = 0, the proposed structure can be considered a bilayer slab structure. It can be seen in Figure 5a that regardless of whether LCP or RCP waves were incidents, the TM wave component in the transmitted wave decreased with increasing rotation angle, whereas the TE wave component gradually increased. However, the overall TE wave component was low; therefore, the TM wave component played a major role in spin thermal radiation at this time. Thus, CD mainly originated from the difference in TM wave components in the transmitted waves at the incidence of LCP and RCP waves. Clearly, the difference between TM wave components in the transmitted wave for LCP and RCP incidence was small at any rotation angle. Combined with Figure 4a, it was found that CD was always at a low level at *f* = 0, which coincides with the result in Figure 5a. The phenomenon in Figure 5b is more obvious in Figure 5a; *f* = 0.6, *d*_1_ = 4.8 μm and *d*_2_ = 0.4 μm. TE wave components of LCP and RCP waves were almost zero, whereas the difference in the TM wave components reached a maximum at a rotation angle of 40°, which corresponds almost exactly to when *f* = 0.6 in Figure 4a. These results further indicate that the difference in the TM wave was the key to influencing spin thermal radiation.

**Figure 6:**
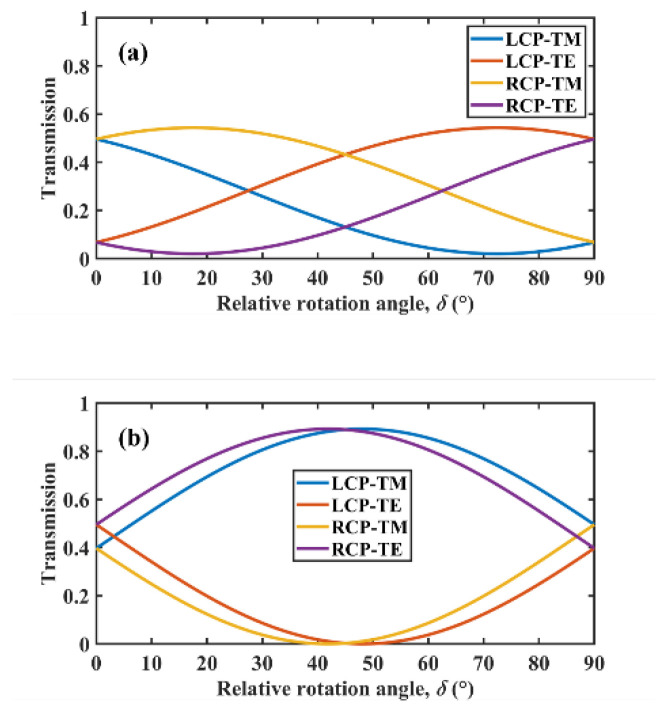
TE wave and TM wave components in the transmitted wave as a function of the rotation angle for LCP and RCP waves: (**a**) single layer slab (*f* = 0) and (**b**) single-layer rectangular strips structure (*f* = 0.6).

To further illustrate the above mechanism, we now discuss the polarization conversion for the monolayer α-MoO_3_ slab, shown in Figure 6. In Figure 6a, it can be seen that differences in TM and TE wave components in the transmitted wave for LCP and RCP waves were basically the same, and both were relatively low overall. When the wavelength was 12 μm, the permittivity of α-MoO_3_ in the *x* and *y* directions were $\varepsilon_{x}=-45.51-7.99i$ and $\varepsilon_{y}=-0.4-0.04i$, respectively. As the real part of $\varepsilon_{x}$ is negative and has a large absolute value, the α-MoO_3_ exhibited metal-like properties in the *x* direction. After quantitative calculation, the transmission was only 0.135 when the TE wave related to $\varepsilon_{x}$ was incident at a 0.175 μm thick monolayer α-MoO_3_ slab, whereas the transmission for the TM wave related to $\varepsilon_{y}$ could reach 0.99. Thus, the effect of the difference in the TM wave component on CD was further confirmed.

Next, the polarization conversion for single-layer rectangular strips was studied. According to the effective medium theory, the permittivity in *x* and *y* directions can be written as $\varepsilon_{x}=1.69-0.004i$ and $\varepsilon_{y}=0.44-0.01i$. Therefore, both TE and TM waves can theoretically be transmitted in a single-layer rectangular strips structure. Figure 6b illustrates that the TM wave component in the transmitted wave for LCP wave incidence tended to increase and then decrease with an increase in the rotation angle, whereas the TM wave component in the transmitted wave for the RCP wave incidence first decreased and then increased. Thus, the TM component was significantly different in the transmitted wave for LCP and RCP waves. However, there was also a large difference in the TE wave component of the transmitted wave at LCP and RCP incidence, which means that the main role of the rectangular strips structure in the top layer was to achieve polarization conversion. According to the polarization conversion results, we then placed the rectangular strips on a 0.4 μm thick substrate and found that the transmission of TE waves was only 0.018, which further indicated that the TE wave component could not pass through the substrate and had little effect on CD. In contrast, the transmission of TM waves could reach 0.969. These results suggest that the role of the substrate was to achieve selective transmission to TE and TM waves. In addition, the trend of the TM wave component difference with rotation angle illustrated in Figure 6b was essentially the same as that in Figure 5b, indicating that the difference in the TM wave component played a decisive role in CD.

## 4. Conclusions

In summary, we systematically investigated the spin thermal radiation in a twisted bilayer α-MoO_3_ metasurface. With the introduction of the filling factor *f*, the spin thermal radiation was greatly enhanced and more flexibly excited. The numerical results show that CD could reach 0.9 via optimizing the filling factor, thickness and rotation angle. Based on analysis of bilayer and single layer structures, it was found that the spin thermal radiation of the structure originated from the polarization conversion of the top periodic rectangular strips structure and the selective transmission of the substrate. Specifically, the difference in the TM wave component of the transmitted wave for LCP and RCP waves incidence effected the structure’s CD. The TM wave component in the transmitted wave was affected by the filling factor; therefore, the spin thermal radiation of the structure proposed in this paper could be flexibly tuned by the filling factor. We believe that this study has potential applications in biosensing and radiation detection.

## Figures and Tables

**Table 1:** Values and parameters of the permittivity.

Physical Parameter	Value	Physical Parameter	Value
$\varepsilon_{\infty ,x}$	4	$\omega_{TO,x}^{}$	1.5457×10^14^ rad/s
$\varepsilon_{\infty ,y}$	5.2	$\omega_{\mathrm{TO},y}$	1.8322×10^14^ rad/s
$\varepsilon_{\infty ,z}$	2.4	$\omega_{TO,z}^{}$	1.8058×10^14^ rad/s
$\omega_{LO,x}^{}$	1.8322×10^14^ rad/s	$\Gamma_{x}$	7.5398×10^11^ rad/s
$\omega_{LO,y}^{}$	1.6041×10^14^ rad/s	$\Gamma_{y}$	7.5398×10^11^ rad/s
$\omega_{LO,z}^{}$	1.8925×10^14^ rad/s	$\Gamma_{z}$	3.7699×10^11^ rad/s

## Data Availability

Data presented in this study are available on request from the corresponding author.
